# Molecular Neuropathology of Astrocytes and Oligodendrocytes in Alcohol Use Disorders

**DOI:** 10.3389/fnmol.2018.00078

**Published:** 2018-03-20

**Authors:** José J. Miguel-Hidalgo

**Affiliations:** Department of Psychiatry and Human Behavior, University of Mississippi Medical Center, Jackson, MS, United States

**Keywords:** glia, alcoholism, postmortem, human, animal models, molecular markers, myelin, epigenetic

## Abstract

Postmortem studies reveal structural and molecular alterations of astrocytes and oligodendrocytes in both the gray and white matter (GM and WM) of the prefrontal cortex (PFC) in human subjects with chronic alcohol abuse or dependence. These glial cellular changes appear to parallel and may largely explain structural and functional alterations detected using neuroimaging techniques in subjects with alcohol use disorders (AUDs). Moreover, due to the crucial roles of astrocytes and oligodendrocytes in neurotransmission and signal conduction, these cells are very likely major players in the molecular mechanisms underpinning alcoholism-related connectivity disturbances between the PFC and relevant interconnecting brain regions. The glia-mediated etiology of alcohol-related brain damage is likely multifactorial since metabolic, hormonal, hepatic and hemodynamic factors as well as direct actions of ethanol or its metabolites have the potential to disrupt distinct aspects of glial neurobiology. Studies in animal models of alcoholism and postmortem human brains have identified astrocyte markers altered in response to significant exposures to ethanol or during alcohol withdrawal, such as gap-junction proteins, glutamate transporters or enzymes related to glutamate and gamma-aminobutyric acid (GABA) metabolism. Changes in these proteins and their regulatory pathways would not only cause GM neuronal dysfunction, but also disturbances in the ability of WM axons to convey impulses. In addition, alcoholism alters the expression of astrocyte and myelin proteins and of oligodendrocyte transcription factors important for the maintenance and plasticity of myelin sheaths in WM and GM. These changes are concomitant with epigenetic DNA and histone modifications as well as alterations in regulatory microRNAs (miRNAs) that likely cause profound disturbances of gene expression and protein translation. Knowledge is also available about interactions between astrocytes and oligodendrocytes not only at the Nodes of Ranvier (NR), but also in gap junction-based astrocyte-oligodendrocyte contacts and other forms of cell-to-cell communication now understood to be critical for the maintenance and formation of myelin. Close interactions between astrocytes and oligodendrocytes also suggest that therapies for alcoholism based on a specific glial cell type pathology will require a better understanding of molecular interactions between different cell types, as well as considering the possibility of using combined molecular approaches for more effective therapies.

## Introduction

Alcohol use disorder (AUD) is defined in the webpage of the National Institute of Alcohol Abuse and Alcoholism as a chronic relapsing brain disease involving compulsive alcohol use, loss of control over alcohol intake, and a negative emotional state when not using. This disorder results in severe behavioral, neurological and other medical pathologies that eventually depend on disturbances of cellular function and metabolism (Abrahao et al., 2017). In the nervous system, these disturbances cause abnormal exchanges of information between brain centers, including cortical and subcortical regions that control the intake of rewarding substances such as alcohol itself, or regions involved in emotional and cognitive regulation (Moselhy et al., 2001; Schulte et al., 2010). In many AUD patients, alcohol-induced brain alterations are also reflected in structural damage in the gray and white matter (GM and WM; Rosenbloom et al., 2003; Zahr and Pfefferbaum, 2017).

It is well known that a large proportion of alcoholics, despite serious behavioral and emotional pathology, show only minor or inconspicuous neurological deficits of the kind mentioned above, and thus these subjects are dubbed as “uncomplicated” alcoholics. In these subjects, AUDs do not necessarily result in catastrophic or global loss of brain tissue of neuronal or glial cell numbers (Jensen and Pakkenberg, 1993). However, application of conventional MRI imaging techniques has shown that even in these AUD subjects atrophy of the cerebral GM volume at relatively younger ages, while later in life the reduction in volume extends to the WM and cerebral ventricles (Pfefferbaum et al., 1988, 1992). This progressive decline in GM volume was first described as affecting the brain globally, but further neuroimaging investigations have demonstrated that significant age-dependent volume reduction is particularly noticeable in the prefrontal cortex (PFC; Pfefferbaum et al., 1997). Furthermore, while only macroscopic volumetric variations can be safely assessed in brain tissue with conventional MRI, diffusion tensor imaging (DTI) studies, a more recent development of MRI with increased resolution to visualize fiber bundle structure in WM, have shown that WM volume changes parallel structural alterations in specific WM axon bundles connecting PFC to brain circuits involved in reward and emotion regulation (Schulte et al., 2010) or that, even when macrostructural changes are not patent, there could be significant microstructural disturbances of axon bundles (Pfefferbaum and Sullivan, 2002). MRI- and DTI-based detection of volumetric and fiber bundle alterations most likely betray disturbed signal processing in specific brain regions such as the PFC and the hippocampus and anomalous connectivity between those brain regions. In fact, uncomplicated subjects are not entirely free of neuropathological alterations at the cellular level either because they show regionally selective degeneration of pyramidal neurons or their dendrites in some brain regions such as the PFC (Kril et al., 1997).

### Role of Glial Cells in AUDs

Since neurons are the main conveyors of information between brain regions, much attention has been placed on the role of neuronal pathology in the various disorders caused by AUDs in human subjects and experimental animals (Abrahao et al., 2017). However, it is well-known that maintenance, survival and normal activity of neurons are fully dependent on the interaction with several types of glial cells (Barres, 2008). These cells assist critically in the support of neurotransmission, propagation of action potentials, survival (of neurons and other glial cells), supply of metabolites, brain injury repair, neuroprotection and synapse formation and removal. In the central nervous system, the main classes of glial cells are astrocytes, oligodendrocytes, NG2 cells and microglia. Each of these cell types identifies mainly with one or two of the support functions mentioned (for instance, astrocytes with neurotransmitter reuptake and metabolic support, oligodendrocytes with myelin formation around axons, microglia with responses to injury and repair), but some functions are performed cooperatively by two or more cell types. Thus, if prolonged alcohol exposure damages glial cells or disrupts their activity, grave disturbances of neuronal function are to be expected. Conversely, due to the existence of neuronal signals that regulate glial physiology (Haydon, 2000; Simons and Trajkovic, 2006), indirect actions of alcohol, mediated by neuronal pathology, are to be expected on the structural and functional integrity of glial cells. Many studies have shown that alcohol exposure *in vivo* and *in vitro* profoundly affects the development, morphology, physiology and gene expression of astrocytes, oligodendrocytes, microglia and NG2 cells. The effects of AUDs on oligodendrocytes were some of the first to receive attention from clinicians and investigators because alcoholism leads to severe neurological and cognitive disorders associated with myelin pathology (Sun et al., 1979; Gallucci et al., 1989; Harper, 2009). Later developments have also shown that the development, physiology, gene expression and morphology of astrocytes are profoundly affected by alcohol abuse (Kennedy and Mukerji, 1986; Renau-Piqueras et al., 1989; Cullen and Halliday, 1994; Franke, 1995).

Many reviews and original research articles have dealt with specific morphology, molecular markers and functions that characterize classes and types of astrocytes (Rajan and McKay, 1998; Laming et al., 2000; Nedergaard et al., 2003; Oberheim et al., 2006; Takano et al., 2006; Zhang and Barres, 2010; Lovatt et al., 2012; Parpura et al., 2012; Kettenmann et al., 2013), oligodendrocytes (Cahoy et al., 2008; Wegner, 2008; Emery and Lu, 2015; Fitzpatrick et al., 2015; Simons and Nave, 2015; Purger et al., 2016; Snaidero and Simons, 2017) and the other glial cell classes in the brain of vertebrates, including the human brain. In this review, I will concentrate on astrocytes and oligodendrocytes in the brain WM and cortical GM mainly because they are the primary glial cell types implicated in the integration (astrocytes) and propagation (oligodendrocytes) of neural signals originating from and arriving in the cortex and the most extensively studied regarding AUDs. I refer to the cited reviews and original articles for more detailed information on normal development, structure, molecular biology and physiology of astrocytes, oligodendrocytes and related cell subtypes. Likewise, the present review is about the glial molecular pathology in AUDs in the context of postmortem and neuroimaging studies, and does not include a detailed discussion of glial pathology in fetal alcohol spectrum disorders (FASD), although occasional reference to FASD is made to illustrate some general points about the pathology of astrocytes or oligodendrocytes in alcohol abuse disorders.

## Astrocytes

### Diversity of Astrocytes

Since the earliest structural and developmental studies astrocytes and related cells have been classified into several subtypes according to their localization and morphology (Reichenbach and Wolburg, 2012). In fact, astrocytes that reside in specific neural niches tend to substantially differ from those in other niches. For instance, astrocytes in WM are considered to be mostly of the fibrillary type while most astrocytes in GM display a distinctive morphology and are classified as protoplasmic astrocytes (Reichenbach and Wolburg, 2012). In turn, in some WM tracts, such as the optic nerve, astrocytes are further subdivided into type 1 and type 2 astrocytes, while GM astrocytes in contact with the meningeal pia mater or those adjacent to the ventricular surfaces are also morphologically distinct from the protoplasmic astrocytes (Reichenbach and Wolburg, 2012). In the cerebellum, astrocytes take on a distinctive morphology that parallels the structure of Purkinje cell’s dendrites and are identified as Bergman glia, while retinal astrocytes radially span across the retinal layers and are called Mueller cells (Kettenmann et al., 2013).

In recent years, it has become evident that the morphological variety is matched by an even richer molecular differentiation of astrocytes (Verkhratsky and Nedergaard, 2018), and that even astrocytes considered as a morphologically homogenous type (Cui et al., 2001; Oberheim et al., 2006; Khakh and Sofroniew, 2015; Hu et al., 2016), for example, cerebral cortex and WM astrocytes, can be further subdivided according to their molecular markers or their ability to divide (Zhang and Barres, 2010). These subtype-specific molecular markers ultimately betray a significant degree of physiological differentiation among astrocytes as they adapt to specific neural niches and functions.

### Roles of Astrocytes in Gray and White Matter

The differential features of astrocytes in the cortical GM reflect a multitude of regulatory roles in support of cortical neuronal function (Verkhratsky and Nedergaard, 2018). These roles can be significantly disturbed either by direct actions of ethanol on receptors, transporters or metabolic enzymes of astrocytes, or through indirect actions mediated by the effects of ethanol on neurotransmission and neuronal metabolism (Verkhratsky and Parpura, 2010; Adermark and Bowers, 2016). Prominent among the critical roles of astrocytes are the exchange with blood vessels of energy-rich metabolites to support neuronal metabolism, the buffering of extracellular ions exchanged during synaptic neurotransmission/propagation of action potentials, the reuptake of synaptically-released glutamate and gamma-aminobutyric acid (GABA), the recycling of these and other transmitters for reutilization or metabolism, the release of small molecular cofactors, such as serine or glycine required for activation of the N-methyl-D-aspartate-type (NMDA-type) glutamate receptors, the release of gliotransmitters or cytokine-like molecules to communicate with other neural cells, and the expression of immune-like activities within the nervous system (Cali et al., 2008). This variety of neurophysiological roles is made possible by abundant expression of specific proteins and molecules and their associated intracellular pathways, which are in many cases exclusive to or predominantly expressed in astrocytes (Verkhratsky and Nedergaard, 2018).

Many of their characteristic intracellular pathways are found in all astrocyte subtypes. However, a division of labor also exists among astrocytes such that, for instance, some astrocytes express high levels of excitatory amino acid transporter 1 (EAAT1), other astrocytes are richer in EAAT2, or others, such as Bergmann glia express EAAT5. Most WM astrocytes and those adjacent to the pia mater express high amounts of the cytoskeletal protein glial fibrillary acidic protein (GFAP). In contrast, astrocytes in the middle layers of the cortex, while rich in glutamate transporters have very low levels of GFAP, even if dramatic increases of GFAP can occur in astrocytes after brain injury, toxicity or ischemia.

In addition to their distinctive morphology, WM astrocytes constitutionally express high levels of GFAP (levels that are significantly lower in many GM astrocytes). Even if neuron to neuron synapses are very low in WM, WM astrocytes express glutamate and glutamine transporters (Banner et al., 2002; Miguel-Hidalgo et al., 2010; Roberts et al., 2014). Unlike most GM astrocytes, WM astrocytes and astrocytes in myelinated parts of the GM, are in close contact with oligodendrocyte processes and the outermost layer of myelin, and often form gap junctions with oligodendrocyte cell membranes to allow for direct communication between the astrocyte and oligodendrocyte cytoplasms (Nualart-Marti et al., 2013). The molecular composition of these junctions is connexin subtype-specific, because connexin 43 (Cx43) and Cx30, which are contained on the astrocyte side of the gap junction, are respectively matched with Cx47 and Cx32 contained in the oligodendrocyte cell membrane to form Cx43–Cx47 and Cx30–Cx32 gap junctions (Nagy and Rash, 2000; Orthmann-Murphy et al., 2008). The importance of these astrocyte-oligodendrocyte gap junctions to establishment of myelin has been confirmed in recent studies showing that absence or downregulation of astrocytic Cx43 and Cx30 (or oligodendrocyte Cx47 and Cx32) in transgenic mice models results in major disruption of myelin formation and maintenance, as well as behavioral deficits (Lutz et al., 2009; Magnotti et al., 2011; Wasseff and Scherer, 2011). In addition, the end-feet of some processes of astrocytes reach to most of the nodes of Ranvier (NR) in the CNS. Despite the close and frequent association of these processes with NR, their function is still unknown, although some researchers have proposed that astrocytes and their perinodal processes play an important role in potassium buffering or in the stabilization and organization of myelin formation at the nodes (Black and Waxman, 1988; Kalsi et al., 2004; Serwanski et al., 2017).

End-feet of astrocytic processes also closely abut the basal lamina surrounding endothelial cells of small blood vessels. These contacts around blood vessels are important in the regulation of water movements from the blood circulation and the maintenance and function of the brain blood barrier (Paemeleire, 2002; Simard et al., 2003; Abbott et al., 2006).

## Molecular Pathology of Astrocytes in Alcohol Use Disorders

### Alcohol-Related Neuropathology of Astrocytes

Chronic alcohol exposure induces atrophic features in glial cells, including astrocytes and their precursors in GM (Miguel-Hidalgo and Rajkowska, 2003). Furthermore, alcoholism associates with downregulation of astrocyte specific genes, particularly in subjects with hepatic pathology (Liu et al., 2006). The atrophic changes in astrocytes appear to preferentially occur in certain brain regions, such as PFC and the hippocampus, where alcohol exposure results in reduced numbers or packing density in the general population of glial cells and in astrocytes (as identified by morphological features or GFAP labeling; Korbo, 1999; Miguel-Hidalgo et al., 2002, 2006). In addition, the expression of astrocyte-related genes is significantly downregulated (Liu et al., 2006). In some subjects, chronic AUDs are associated with persistent nutritional deficiencies or hepatic damage, resulting in serious neurological disorders such as Wernicke’s encephalopathy, hepatic encephalopathy or the demyelinating disorders central pontine myelinolysis and Marchiafava-Bignami syndrome (de la Monte and Kril, 2014; Verkhratsky et al., 2014). In these cases, severe neurological symptoms correlate with macroscopically apparent, substantial degeneration of GM or WM in cerebellum, thalamus, mammillary bodies or cerebral cortex (Phillips et al., 1990; Kril and Harper, 2012). Interestingly, although neurons and oligodendrocytes are considered major targets of nutritional and metabolic disturbances, such as thiamine deficiency, astrocytes can be also critically affected by the same disturbances (Hazell, 2009). In addition, acute ethanol exposure of cultured astrocytes causes extensive gene expression changes that resemble the heat shock response (Pignataro et al., 2013).

As it could be expected, the response of astrocytes to pathogenic alcohol exposure is not limited to alterations in their number, morphology or development, but affects many of the roles played by astrocytes in the nervous system. These roles include the regulation of neuroinflammatory processes, calcium signaling, balance of excitatory and inhibitory neurotransmission, water balance/cell volume regulation, as well as the regulation of dopamine-dependent behavioral processes in brain reward circuits (Adermark and Bowers, 2016). In addition, acute or prolonged exposure of astrocytes to alcohol may substantially modify the efficacy of connections between brain areas by disturbing the maintenance of myelin (Hazell, 2009) and the buffering of ions in the proximity of nodes Ranvier. Altering ion buffering and the osmotic regulation that results from astrocyte interactions with oligodendrocytes around NR causes abnormal action potential propagation in WM and myelinated portions of GM (Gankam Kengne et al., 2011).

The alcoholism-related changes in astrocyte numbers and their markers may be due, at least partly, to direct inhibitory effects of ethanol on astrocyte proliferation or turnover. Both in astrocytes cultured from neonatal rodents (Davies and Cox, 1991; Guerri and Renau-Piqueras, 1997; Guerri, 1998) or from postmortem human brain tissue autopsies (Kane et al., 1996) ethanol exposure causes substantial inhibition of astrocyte proliferation and synthesis of DNA and protein, including reduced expression of the major astrocyte marker GFAP. After prolonged exposure, this inhibition may lead to decreased astrocyte numbers, as well as impaired ability to perform the critical functions enumerated earlier in this review. The responses of astrocytes to chronic alcohol, although mostly inhibitory, may also lead to secondary activation of gliosis-like astrocyte responses when AUDs prolong sufficiently into senescence (Miguel-Hidalgo and Rajkowska, 2003; Miguel-Hidalgo, 2009). In approaching late-age, accumulation of ethanol-related deficits in astrocyte structure and function may contribute to neuronal degeneration, and this degeneration would trigger a secondary activation of astrocyte reactivity or gliosis, which would be reflected in increased GFAP production and other gliotic changes, although it is still unclear how other markers of astrocytes respond to aging-associated neuronal degeneration.

### Alcohol Effects on Glutamate Receptors and Astrocyte Components of the Cycle for Release and Reuptake of Glutamate

#### NMDA-Type Glutamate Receptors in AUDs

In patients with AUDs there is evidence, some of it controversial, of alcohol-related dysfunction in some aspects of glutamatergic neurotransmission such as an increase in the expression of NMDA-type glutamate receptors and a decrease in GABA receptors, particularly in chronic alcoholism (Davis and Wu, 2001). Ethanol acts antagonistically at NMDA receptors by reducing their activation by glutamate. In animal models, chronic alcohol consumption increases expression of the NR2A, NR2B and NR1 subunits of NMDA receptors in the neocortex and the hippocampus (Gass and Olive, 2008), and the increases would appear to explain the neuronal hyperexcitability found in animal models after alcohol withdrawal. Although less consistently, human studies in chronic alcoholics also show increases in ligand binding to NMDA receptors (Tsai, 1998; Tsai et al., 1998) mainly in the PFC, but not in other cortical or brain regions such as the cingulate cortex, hippocampus or cerebellar vermis (Freund and Anderson, 1996, 1999). mRNA levels for the same NMDAR subunits as above do not differ in uncomplicated alcoholics as compared to controls, but are reduced in cirrhotic alcoholics (Ridge et al., 2008).

#### Involvement of Astrocyte Glutamate Transporters and Glutamine Synthetase in AUDs

Regardless of how persistent glutamate receptor changes are in human chronic alcoholism, the antagonism of NMDA receptors and the potentiation of GABA receptors by ethanol are likely to alter glutamate release and the concentrations of glutamate in the extracellular space. These neurotransmitter alterations would cause the involvement of glutamate transporters, glutamate converting enzymes and glutamate receptors, which are highly expressed by astrocytes. In the cerebral cortex, the glutamate transporters of astrocytes are crucial to the synaptic reuptake of glutamate. In addition, astrocytic GABA transporters make a very important contribution to GABA reuptake. These transporters, together with the enzyme glutamine synthetase (GS) in astrocytes, are essential components of the cycle that terminates the actions of released glutamate or GABA at many synapses, and allows for further recycling and synaptic release of glutamate and GABA. Thus, some studies have determined the cortical expression of astrocytic glutamate transporter as well as GS mRNA and protein in AUD subjects (Miguel-Hidalgo et al., 2010; Ayers-Ringler et al., 2016). Interestingly, these studies have not detected significant variations in the protein levels of either EAAT1, EAAT2 or GS in alcohol dependence, and some studies *in vitro* actually show that ethanol exposure may cause an increase in the rate of glutamate transport per astrocyte (Smith and Zsigo, 1996; Smith, 1997; Zink et al., 2004), although in some rat brain areas such the nucleus accumbens there appears to be an ethanol-induced decrease in glutamate transport that still is not linked to a reduced expression of glutamate transporters (Melendez et al., 2005).

It is possible that homeostatic mechanisms result in unaltered or increased expression of glutamate receptors or transporters in many brain regions of human alcoholics. However, it must be kept in mind that the unchanged transporter levels we found in the orbitofrontal cortex in alcohol-dependent subjects occurred only in uncomplicated alcoholism, while in subjects with comorbid major depression there were reduced levels of glutamate transporters and GS, raising the possibility that the severity of alcohol-related pathology resulting in depression involves a decrease in the astrocytic components of the glutamate cycle (Miguel-Hidalgo et al., 2010). The loss of glutamate transporters in subjects with Wernicke’s encephalopathy, often associated with severe cases of chronic alcoholism (Hazell et al., 2010) may be considered further evidence for the view that the severity of pathology in some brain areas may depend on profound changes in the glutamate cycle components of astrocytes.

Animal studies have shown that different regimes of chronic alcohol intake, withdrawal, and reinstatement have diverse effects on the expression of glutamate cycle components. This diversity raises the possibility that different trajectories in the timing, length and frequency of withdrawal periods, or the comorbidity with other disorders cause ample variation in glutamate-related mRNA and protein markers in human alcoholics at the time of death. This variety would prevent finding statistically significant differences in AUD patients as compared to non-alcoholic subjects. In fact, as mentioned above, we found that among subjects with alcohol-dependence only those with a comorbid diagnosis of depression had significantly lower levels of glutamate transporters EAAT1 (and a tendency for lower EAAT2 transporters) than controls, while in alcoholics without other psychiatric diagnoses (Miguel-Hidalgo et al., 2010) there was no change, suggesting that uncomplicated AUDs may involve compensatory regulation of glutamate transport. In line with this suggestion, some animal models of alcoholism show increase in astrocytic glutamate transporter levels (Wu et al., 2011), even if alcohol itself can disrupt the function of those transporters (Mulholland et al., 2009). Interestingly, restoration of EAAT2-based glutamate transport with ceftriaxone actually reduces alcohol drinking (Lee et al., 2013), while the reduction of astrocytic EAAT1 resulting from deletion of the circadian period gene (Per2) in mice is accompanied by increased alcohol intake (Spanagel et al., 2005).

#### Astrocyte-Released NMDA Receptor Co-Agonists in AUDs

Regulation of glutamatergic transmission at NMDA receptors is also dependent on glycine, which acts as co-agonist at those receptors. At the same binding-site, astrocyte-released D-serine is also an active co-agonist. Both glycine and D-serine have a permissive role in NMDA receptor activation when binding to the glycine-binding site. Ethanol can compete with D-serine for the occupancy of that site, although the dependence of behavioral sensitivity on ethanol binding is related to the exact subunit composition of the NMDA receptors and thus differs across brain regions (Tsai, 1998). On the other hand, reduced affinity for glycine at the glycine site is positively associated with attenuated sensitivity to the behavioral effects of alcohol (Kiefer et al., 2003) in mice, while tolerance to partial agonists of that site appears to develop in alcohol dependent subjects (Krystal et al., 2011), pointing to an important role of astrocyte-produced D-serine in the effects of ethanol in chronic AUD patients.

### Astrocyte Thrombospondin in AUD-Related Synaptic Alterations

Alcohol-related neuronal dysfunction may also depend on regressive changes at synaptic contacts that result from intermittent or prolonged alcohol exposure. Those changes may be due, at least partly, to impairments in the ability of astrocytes to provide factors involved in synapse maintenance such as thrombospondins and their receptors (Ullian et al., 2004). In animal models, alcohol exposure results in significant reduction of thrombospondin release that can persist for 24 days, in parallel with disturbed matching of presynaptic and postsynaptic structures (Risher et al., 2015). Hepatic damage caused by alcoholism in some subjects may also result in synaptic dysfunction indirectly mediated by astrocytes, because increased ammonia levels that follow liver dysfunction diminish thrombospondin secretion by astrocytes and reduce the levels of synaptic proteins (Jayakumar et al., 2014). Alcohol exposure during early or prenatal stages of development, and maybe later too, may cause persistent changes in synapse formation involving thrombospondin as well (Trindade et al., 2016). These changes are further accompanied by marked reductions in astrocyte-secreted extracellular matrix (ECM) proteins, such as laminin or heparan-sulfate proteoglycan (Lasek, 2016; Trindade et al., 2016). Thus, repeated alcohol exposure at different stages of prenatal and postnatal development would result in abnormal regulation of astrocyte-derived factors involved in synaptogenesis.

### Astrocyte Processes at the Blood-Brain Barrier and the Involvement of Aquaporins in AUD-Related Neuropathology

Astrocytes processes abut the basal lamina surrounding the endothelial cells of small blood vessels, where they contribute to blood-brain barrier (BBB) maintenance (Prat et al., 2001). In addition, those processes are essential to the exchange of metabolic substrates with the blood circulation, and to the regulation of blood flow (Koehler et al., 2009). Chronic alcoholism disturbs components of the BBB at endothelial cells (Haorah et al., 2005; Rubio-Araiz et al., 2017), impairing to varying degrees the exchange of energy and neurotrophic metabolites such as glucose, that directly impinge on neuronal and glial function (Abdul Muneer et al., 2011a). In addition, alcohol causes direct inhibitory effects on glucose uptake by astrocytes processes (Abdul Muneer et al., 2011b).

Effects of alcohol mediated by astrocytes very likely involve changes in aquaporins as well (Kong et al., 2013), in particular aquaporin 4 (AQ4), a membrane protein highly expressed in astrocytes processes at the BBB that allows effective passage of water through the cell membrane (Badaut et al., 2002; Rajkowska et al., 2013). Repeated alcohol intake in binging rat models results in significant increases in aquaporin, astrocyte swelling (linked to brain edema) and activation of neuroinflammatory cascades (Collins and Neafsey, 2012; Collins et al., 2013). Anti-inflammatory treatments can prevent the effects of the AQ4 elevation that is concomitant with increases in neuroinflammatory markers (Tajuddin et al., 2014). On the other hand, serious consequences of alcoholism, such as the loss of myelin in central pontine myelinolysis, might be associated with reduction in astrocytic aquaporins, although more research seems to be needed to fully ascertain this possibility (Popescu et al., 2013).

In summary, multifaceted actions of ethanol on astrocytic markers involved in the formation and regulation of the BBB, amplified by their reciprocal interactions with the BBB, may be important determinants of the cellular and functional pathology of alcoholism.

## Oligodendrocytes

### Role of Oligodendrocytes in Gray and White Matter

The main function of oligodendrocytes is the formation and maintenance of myelin, which consists of tightly piled layers of oligodendrocyte cell membrane wrapped around the axons of neurons (Baumann and Pham-Dinh, 2001; Butt, 2005). The layers of myelin act as insulation against the dissipation of ionic gradients, allowing for fast, self-regenerating saltatory conduction of action potentials between consecutive NR along the axons to reach synaptic terminals, and thus for efficient exchange of neural impulses between brain centers (Nave and Werner, 2014). Despite this unity of purpose, most oligodendrocytes and their myelin sheaths are finely tuned and sensitive to the physiological and gene expression changes in the neurons whose axons they wrap and the astrocytes that surround them (Simons and Trajkovic, 2006; Nave and Werner, 2014). Conversely, oligodendrocytes produce signals and growth factors that support the axons they enwrap and the neurons and astrocytes in their vicinity (Du and Dreyfus, 2002; Nave and Trapp, 2008; Simons and Nave, 2015). Some oligodendrocytes in GM regions are intimately associated with neuronal cell bodies, but their functions remain so far unclear.

## Molecular Pathology of Oligodendrocytes in Alcohol Use Disorders

### Myelin Components

Postmortem cellular and *in vivo* neuroimaging studies in human subjects have revealed that prolonged and repeated alcohol intake results in various degrees of damage or adaptations in the myelin that sheaths axons in the WM and GM, as well as in the oligodendrocytes that form the myelin. In some subjects, macroscopic damage to the WM caused by alcoholism is apparent, and can be identified with loss of myelin both in neuroimaging and postmortem histochemical studies. WM and GM damage produces different neurological syndromes that can be distinguished according to the specific anatomical location and the nature of the neurological disturbances. Myelin disorders such as Marchiafava-Bignami disease, Wernicke-Korsakoff syndrome, hepatic encephalopathy, central pontine myelinolysis or alcohol cerebellar degeneration involve myelin losses in WM and GM of cortical and subcortical brain regions (Zahr and Pfefferbaum, 2017). In these disorders, BBB disruption or nutritional deficits, such as thiamine deficiency, alone or most likely in interaction with direct effects of alcohol on oligodendrocytes, are considered main culprits for myelin disturbances in chronic alcoholism (Lewohl et al., 2005; Alexander-Kaufman et al., 2007; He et al., 2007). On the other hand, given the frequent co-abuse of ethanol and tobacco, part of the deleterious effects of ethanol on myelin proteins might result from an interaction of ethanol with specific components of tobacco, such as nicotine-specific nitrosamine ketone (NNK; Tong et al., 2015; Zabala et al., 2015; Papp-Peka et al., 2017). However, even in subjects without such obvious neurological and anatomical complications (the “uncomplicated cases”), the expression of myelin and oligodendrocyte-related proteins, or their mRNAs, can be significantly altered in various brain regions, being particularly prominent in the PFC (Mayfield et al., 2002; Alexander-Kaufman et al., 2006; Liu et al., 2006), which are reflected in low levels of the main structural myelin proteins such as myelin basic protein (MBP) and possibly proteolipid protein (PLP), their companions, myelin associated-glycoprotein (MAG) and oligodendrocyte-myelin glycoprotein (Omgp; Okamoto et al., 2006), and related transcription factors (Miguel-Hidalgo et al., 2017). Reduced expression of major myelin proteins such as MBP has been detected in models of prenatal alcohol exposure (Ozer et al., 2000; Bichenkov and Ellingson, 2001), while *in vitro* studies have shown that the effects of ethanol on the expression of myelin components could be mediated by direct regulation of PKC-like enzymes rather than by altering their expression (Bichenkov and Ellingson, 2002).

Beside disturbing the expression of myelin proteins, alcohol can induce oligodendrocyte apoptosis during prenatal development in primates such as the macaque (Creeley et al., 2013), while during early postnatal mice development (roughly equivalent to the human third gestation trimester) there is a dramatic reduction in differentiated oligodendrocytes and oligodendrocyte progenitor cells in the corpus callosum. These cell populations recover after ceasing alcohol exposure, but deficits in MBP levels or in the structure of corpus callosum fibers persist in young adult mice (Saito et al., 2016; Newville et al., 2017). In adult mice, chronic intermittent ethanol (CIE) exposure causes also significant reduction in the levels of MBP, PLP and 2′,3′-cyclic-nucleotide 3′-phosphodiesterase (CNPase) in several brain regions (Samantaray et al., 2015). These degenerative effects appear to be mediated by the calcium-activated protease calpain, because calpain inhibitors prevent the reductions of myelin-related proteins in the mice CIE model (Samantaray et al., 2015).

### Gene Expression in Oligodendrocytes

In human postmortem brain, gene-expression studies of chronic alcoholism that involve the aggregate of GM and WM from frontal cerebral regions, have found significantly decreased mRNAs of myelin-related proteins in chronic alcoholics (Lewohl et al., 2000, 2001; Liu et al., 2006; Farris et al., 2015a). Low myelin-related mRNAs include those of the major structural myelin proteins MBP, PLP, myelin oligodendrocyte glycoprotein (MOG) and MAG. These reductions are particularly significant in chronic alcoholics with cirrhosis as compared to non-alcoholic controls or to non-cirrhotic alcoholics (Lewohl et al., 2005), suggesting that nutritional deficiencies or metabolic toxicity, possibly interacting with direct ethanol effects, strongly deplete the expression of myelin components in alcoholics, at least as assessed in studies that include GM in the probed tissue. In contrast, in a recent study from our laboratory, we used samples only from the WM adjacent to cortical area 47 (orbitofrontal cortex) in chronic alcoholics, and observed that mRNA levels for myelin proteins PLP, MAG and MOG and other oligodendrocyte markers were markedly lower than in controls, although the levels of MBP mRNA were not changed (Miguel-Hidalgo et al., 2017). Since cirrhosis had not been diagnosed in most subjects of our study, these results suggest that the effects of prolonged alcohol abuse in some regions of WM may occur without cirrhosis and be different from those when GM is included. However, the degree of hepatic compromise was not exactly quantified in our samples, so that it was not yet possible to separately assess indirect from direct effects of ethanol on the strong decreases in the expression of mRNAs for some myelin proteins. Despite the highly localized nature of our WM study (all samples were from WM adjacent to Brodmann’s cortical area 47), it is also important to note that factors related to hepatic pathology, GM contamination of samples, or RNA quality may influence changes detected in previous studies of myelin-related markers in alcoholism (Sutherland et al., 2014) and thus replication studies with well-defined locales, or in other brain areas, appear to be necessary to draw the right conclusions regarding effects of chronic alcoholism on gene expression of glial cells in the human brain. A recent study in the hippocampus of human chronic alcoholics has in fact revealed significant decreases in several genes related to myelination in addition to alterations in specific proteins of stress-related pathways that operate in astrocytes (McClintick et al., 2013).

Studies with oligodendrocyte cultures indicate that ethanol-induced degeneration or impairment of myelin maintenance may be mediated by the ethanol metabolite acetaldehyde (Coutts and Harrison, 2015), suggesting that the ability to degrade or eliminate this metabolite may influence the effects of alcohol on myelin composition. Moreover, human subjects with alcoholism most probably differ in their drinking schedules and exposure to binge or withdrawal periods. These periods, according to the results of animal experiments, can significantly alter myelin protein expression (for example causing a recovery of MBP expression; Kipp et al., 2012; Navarro and Mandyam, 2015) and strongly influence mRNA levels at the time of death. Summarizing, despite complex interactions that probably determine the individual levels of myelin-related mRNAs and proteins at the time of death, the available animal experimentation and human postmortem evidence indicates significant effects of ethanol abuse on the expression of myelin components, and the plasticity of myelin itself. These changes would significantly affect the role of myelin maintenance in action potential propagation.

### Oligodendrocyte Survival and Proliferation

In addition to the effects on myelin components and structure, ethanol exposure very likely causes damage to oligodendrocyte precursors by reducing their proliferation (Newville et al., 2017) or disrupting the expression of transcription factors, such as c-fos (Bichenkov and Ellingson, 2009), that regulate the differentiation of those precursors into mature, myelin-forming oligodendrocytes. The effects on differentiation may also include abnormal acceleration of oligodendrocyte differentiation (Aspberg and Tottmar, 1994). However, at a difference with animal models of prolonged alcohol exposure, chronic alcoholism in humans may not necessarily cause an overall reduction in neuro- or glio-genesis in well-known neurogenic niches (Sutherland et al., 2013). On the other hand, a direct role of ethanol in promoting myelin pathology and the possibility of recovery from that pathology are strongly suggested by increased MBP levels in the medial PFC of rats after prolonged periods of abstinence from ethanol (Navarro and Mandyam, 2015). Potential for recovery appears to be a consequence of the involvement of oligodendrocyte precursor cells in remyelination processes (Mi et al., 2009) during abstinence from alcohol drinking.

## Glia-Related Extracellular Matrix Components in AUDs

In WM, several proteoglycans and other ECM proteins are produced by astrocytes, oligodendrocytes and neurons to form adhesion complexes at the NR and axon initial segments, where they are implicated in the aggregation of voltage-gated sodium channels and other components involved in action potential generation and propagation (Zimmermann and Dours-Zimmermann, 2008; Nelson and Jenkins, 2017). Those complexes include brevican, versicans, neurocan, tenascins and neurofascins, among others, form mutual attachments involving interactions between specific protein domains. Interestingly, repetitive binge alcohol intake in adolescent rats significantly increases the levels of several of those proteins in the WM of brain areas such as the orbitofrontal cortex that participate in the pathophysiology of addictive behaviors (Coleman et al., 2014). Alcohol exposure also significantly alters the production by astrocytes and oligodendrocytes of some ECM proteins involved in the formation of perineuronal nets and synapses in the GM, and in the assembly of the basal lamina around blood vessels (Lasek, 2016), although the exact role of those changes in the mechanisms leading to behavioral and functional disturbances in AUDs is not fully understood. The importance of an involvement of ECM components in the mechanisms of alcohol addiction is suggested by the ability of ECM disturbances to modulate the seeking for alcohol and other drugs in knock-out mice lacking matrix metalloproteinase 9 (MMP-9), a protease that regulates the integrity of the ECM (Smith, 2017). These mice have reduced motivation for alcohol drinking, but rescuing MMP-9 activity in the brain’s amygdala restores normal alcohol-seeking behavior (Stefaniuk et al., 2017).

## Epigenetic Changes in Oligodendrocytes and Astrocytes in AUDs

Regulation of DNA transcription into mRNA is greatly dependent on epigenetic mechanisms such as DNA methylation and acetylation, and methylation of chromatin histones (Gräff et al., 2011). In addition, at the translational level, gene expression is regulated by the activity of microRNAs (miRNAs), small forms of non-coding RNA (about 22 nucleotides long) that interfere with translation into proteins by binding to specific sequences of coding mRNA (Liu and Casaccia, 2010; Li and Yao, 2012; Emery and Lu, 2015).

In recent years, several reviews have compiled studies showing that the development of astrocytes, oligodendrocytes as well as plastic changes in myelin maintenance involve complex epigenetic pathways (MacDonald and Roskams, 2009; Kim and Rosenfeld, 2010; Yu et al., 2010; Bian et al., 2013; Namihira and Nakashima, 2013; Emery and Lu, 2015). These pathways can be significantly altered by prolonged exposure to alcohol (Aspberg and Tottmar, 1994; Bichenkov and Ellingson, 2009; Alfonso-Loeches et al., 2012; Creeley et al., 2013; Coutts and Harrison, 2015; Newville et al., 2017). Methylation of DNA at specific nucleotides, and acetylation and methylation of DNA-associated histones are known to critically determine the fate and differentiation of precursors into mature astrocytes and oligodendrocytes as well as the formation of myelin (Moyon et al., 2016).

Epigenetic and miRNA-mediated mechanisms in the central nervous system play also relevant roles in the pathophysiology of neurological, neurodegenerative and psychiatric disorders (Meza-Sosa et al., 2012). The clinical relevance of increasing our knowledge about epigenetic disturbances in oligodendrocytes and astrocytes stems from the demonstration of significant epigenetic anomalies in demyelinating disorders, and the possibility of reversing them with experimental treatments targeted to epigenetic alterations (Li and Yao, 2012; Liu et al., 2016). In addition, several miRNAs have been found to regulate directly (by impairing translation) or indirectly (through other miRNAs or transcription factors suppressed by miRNAs) the production of transcription factors and proteins during development (Bian et al., 2013).

### Glial Epigenetic Markers

AUDs are associated with profound brain alterations in epigenetic markers (Zhou et al., 2011; Farris et al., 2015b; Weng et al., 2015; Legastelois et al., 2017) and significant increases in miRNAs regulating the expression of many proteins (Lewohl et al., 2011). Alcohol-related epigenetic changes have been found in DNA methylation patterns and in methylation and acetylation of histones in the human PFC, hippocampus and amygdala (Ponomarev, 2013; Farris et al., 2015b) as well as in cultured astrocytes (Zhang et al., 2014). Some studies suggest that acute alcohol intake leads to histone deacetylase (HDAC) inhibition in the amygdala that would be the basis for increased histone acetylation and anxiolysis, while withdrawal, anxiety or adolescent alcohol exposure seems to be associated with increased HDAC activity (Pandey et al., 2017) and decreased acetylation (Pandey et al., 2008). This HDAC increase would lead to reduced expression of genes involved in synaptic plasticity, and, consequently, HDAC inhibitors have been suggested as a therapeutic option to reduce anxiety and alcohol intake (Pandey et al., 2017). Other researchers have demonstrated DNA methylation disturbances, mostly reductions, in humans with AUDs, although a more recent study with different methodology points to a relatively higher percentage of hypermethylated methylation sites in brain DNA of subjects with alcoholism (Tulisiak et al., 2017).

Methylation changes in the human brain appear to be accompanied by reduction in the mRNA expression of DNA methyltransferases (DNMTs), while in animal models repeated alcohol exposure results in DNMT upregulation (Tulisiak et al., 2017). Actually, the effects of alcoholism on DNA methylation patterns involve both hypo- and hypermethylation in promoters for specific genes, producing a rather complex picture of methylation effects (Tulisiak et al., 2017). However, given that alcohol exposure in animal models increases DNMTs’ levels some researchers have explored DNMT inhibition as a therapeutic approach against excessive alcohol drinking, finding that 5-aza or decitabine, both DNMT inhibitors, acutely reduce excessive alcohol drinking in rats or mice under certain conditions (Ponomarev et al., 2017; Tulisiak et al., 2017). Likewise, developmental models of alcoholism in rodents show disruption of DNA methylation and other epigenetic markers in various regions of the central nervous system (Laufer et al., 2017; Mahnke et al., 2017; Öztürk et al., 2017).

Recent studies on the effects of HDAC and DNMT inhibitors in AUD mouse models have shown that treatment with the DNMT inhibitor decitabine results in decreased ethanol drinking and upregulated expression of genes highly represented in oligodendrocytes and astrocytes in the ventral tegmental area, key region in the brain reward pathways (Ponomarev et al., 2017). In addition, exposure of cultured astrocytes to ethanol results in decreased methylation of the tissue plasminogen activator (TPA) gene promoter (Tulisiak et al., 2017). TPA is involved in ECM degradation, and has been reported to be increased in animal models of AUDs (Zhang et al., 2014). In rats, prenatal ethanol exposure leads to hypermethylation of the promoter for the astrocyte GFAP gene and to reduction in GFAP expression during early postnatal development (Vallés et al., 1997). These findings suggest that alteration of DNA or histone epigenetic markers in astrocytes and oligodendrocytes may play an important role in mediating behavioral disturbances in AUDs.

### Glial miRNA Expression Changes

Some studies have also targeted putative miRNA changes in oligodendrocytes following repeated or prolonged alcohol exposure and the possibility that such alterations may bring about disturbances in myelination. In fact, alcoholism has been found associated with upregulation in the expression of miRNAs from a gene cluster in chromosome 14q32 that have for targets the mRNAs of several proteins involved in processes of oligodendrocyte proliferation and myelination (Manzardo et al., 2013). It is also unsurprising that genome-wide examination of miRNA-protein gene co-expression networks in the brain of alcohol-dependent human subjects has identified abundant epigenetic modifications in molecular networks that operate within oligodendrocytes and astrocytes (Ponomarev et al., 2012).

Studies have also shown significant increases of PFC miRNAs in subjects with AUDs (Lewohl et al., 2011), although in WM some miRNAs would be decreased (Miguel-Hidalgo et al., 2017). Increased miRNAs include some targeting major myelin proteins such as PLP1 and CNPase as well as transcription factor C11ORF9, a regulator of myelin formation. In our recent work, we found that miR-21, high in oligodendrocytes, was strongly and positively correlated with decreased PLP1 miRNA (which is not a direct target of miR-21; Miguel-Hidalgo et al., 2017). The networks and pathways regulated by differentially expressed miRNAs in human alcoholics and mice are very highly enriched in oligodendrocytes and astrocytes, some of them exclusively for each cell type (Nunez et al., 2013). Thus, miRNA increases are consistent with downregulated expression of myelin components and other oligodendrocyte pathology in AUD patients.

## Concluding Remarks

**Figure 1:**
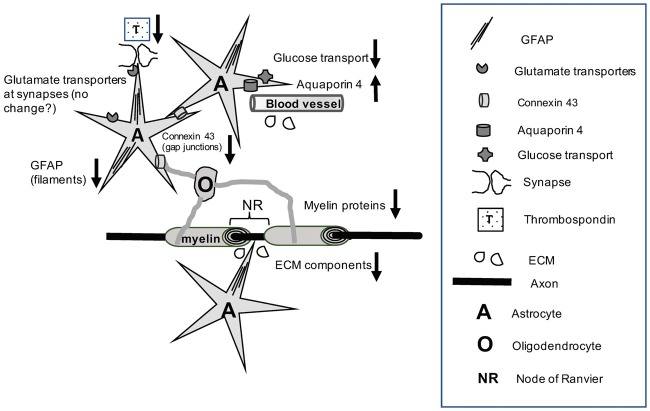
The cartoon illustrates that chronic alcohol exposure results in a variety of molecular disturbances that affect the reciprocal interactions between astrocytes and oligodendrocytes (for example, through reduction of connexin levels), as well as between these glial cells and neurons at synapses, NR or in the wrapping of axons by myelin. Alcohol exposure also results in pathological alterations of astrocyte-derived components at the blood brain barrier (BBB) and the ECM. More recent research work has revealed epigenetic abnormalities or changed levels of specific non-coding RNAs in alcohol use disorders (AUDs). Nevertheless, further research is needed to understand the mechanisms by which alcohol-related functional pathology at the cell and protein expression levels is linked to epigenetic and non-coding RNA markers. Abbreviations: A, astrocytes; ECM, Extracellular matrix; GFAP, glial fibrillary acidic protein; NR, node of Ranvier; O, Oligodendrocyte; T, Thrombospondin; ↓, Down-regulation; ↑, Up-regulation.

Ethanol exposure in AUDs results in disturbances in the structure of astrocytes and oligodendrocytes as well as in the expression and function of specific astrocytes and myelin proteins. The disturbances are likely to impair diverse aspects of neuronal function including regulation of synaptic transmission, synapse formation, metabolism, interactions with the brain blood supply, propagation of action potentials and neuroprotection. Moreover, the complex interaction between astrocytes and oligodendrocytes involves proteins that are affected by ethanol exposure, such as specific connexins at gap junctions, glutamate transporters, or ECM proteins produced by astrocytes, oligodendrocytes and neurons that are crucial for saltatory conduction at NR (Figure 1). However, much more research is needed to determine the mechanisms by which AUDs acting on the components that support the interactions between astrocytes and oligodendrocytes lead to failures in connectivity between brain regions, either by affecting myelin structure or the ability to regenerate action potentials between NR. In addition, chronic alcoholism causes disturbances in the expression of miRNAs and other epigenetic markers that directly influence protein expression. These regulatory changes very likely underpin alterations of proteins and functional pathways in astrocytes and oligodendrocytes observed in earlier studies. However, with a few exceptions, it is still unclear how protein expression changes and the functional pathways they serve in astrocytes and oligodendrocytes depend on non-coding RNA and epigenetic alterations and what is the contribution of these glial processes to the neuronal pathophysiology of alcoholism. In conclusion, much additional work is needed to understand at molecular and neurophysiological levels the mechanisms of alcohol-related neural damage that depend on the molecular pathology of astrocytes, oligodendrocytes and their interactions.

## Author Contributions

JJM-H conceived and wrote the review.

## Conflict of Interest Statement

The author declares that the research was conducted in the absence of any commercial or financial relationships that could be construed as a potential conflict of interest.
